# Efficacy of Treatments Targeting Hypothalamic-Pituitary-Adrenal Systems for Major Depressive Disorder: A Meta-Analysis

**DOI:** 10.3389/fphar.2021.732157

**Published:** 2021-09-10

**Authors:** Yudan Ding, Zirou Wei, Haohao Yan, Wenbin Guo

**Affiliations:** ^1^ National Clinical Research Center for Mental Disorders, Department of Psychiatry, The Second Xiangya Hospital of Central South University, Changsha, China; ^2^ Mental Health Center, The Second Affiliated Hospital, Guangxi Medical University, Nanning, China

**Keywords:** depression, hypothalamic-pituitary-adrenal (HPA) axis, mifepristone, vasopressin 1B receptor antagonist, randomized controlled trials (RCTs), drug effect

## Abstract

Abnormal hypothalamic-pituitary-adrenal (HPA) axis has been implicated in major depressive disorder (MDD). A number of studies have attempted to use HPA-modulating medications to treat depression. However, their results are inconsistent. The efficacy of these drugs for MDD remains uncertain. The aims of this meta-analysis were to determine the effect and safety profile of HPA-targeting medications for MDD. World of Science and PubMed databases were comprehensively searched up to March 2021. All randomized controlled trials (RCTs) and open-label trials exploring antiglucocorticoid and related medications in patients with depression were included. Standardized mean differences (SMDs) and risk ratios (RRs) with 95% confidence intervals (CIs) were calculated for continuous or dichotomous outcomes, respectively. In the meta-analysis, we identified 16 RCTs and seven open-label studies that included 2972 subjects. Pooling the change data that assessed the efficacy across all included HPA-targeting medications for depression showed a significant difference between interventions and controls with very small heterogeneity after influence analysis (SMD = 0.138, 95%CI = 0.052, 0.224, p = 0.002; I^2^ = 20.7%, p = 0.212). No obvious publication bias was observed (p = 0.127). Effectiveness remained significant in patients with MDD (SMD = 0.136, 95%CI = 0.049, 0.223, p = 0.002). Subgroup analysis showed a significant difference favoring mifepristone and vasopressin 1B (V_1B_) receptor antagonist treatment. Adverse events were reported by 14 studies and our analysis of high-quality studies showed a significant difference in favor of controls (RR = 1.283, 95%CI = 1.134, 1.452, p = 0). Our study suggested that patients with MDD may benefit from mifepristone and V_1B_ receptor antagonist treatments that have tolerable side effects. HPA-based medications are promising for depression treatment. However, additional high-quality RCTs, including head-to-head trials, are needed.

**Systematic Review Registration:**https://www.crd.york.ac.uk/PROSPERO/, identifier registration number: CRD42021247279

## Introduction

Major depressive disorder (MDD) is a common and costly mental disorder that is characterized by pervasive low mood and various other symptoms, such as cognitive and physical symptoms. Its probability of recurrence is high, with an average of four episodes during a patient’s life (Limosin et al., 2007). Nearly 30% of individuals with MDD always have symptoms present and develop a chronic condition that is reliant on the illness stage and other risk factors such as childhood trauma and personality (Angst et al., 2009; Boschloo et al., 2014). It substantially affects an individual’s psychosocial functioning and exhausts quality of life (Malhi and Mann, 2018). The Global Burden of disease Study 2019 states that (Diseases and Injuries, 2020) depressive disorders are among the top 10 causes of disability-adjusted life-years (DALYs) for the 10–49-years age group and are among the top three causes of DALYs for women. Barely any breakthrough in the optimization of MDD diagnosis and the improvement of MDD treatment outcomes have been made over the past several decades despite the considerable efforts exerted worldwide. Some novel antidepressants targeting the N-methyl-D-aspartate receptor or gamma-aminobutyric acid are still in the early stages for development or approval. The Sequenced Treatment Alternatives to Relieve Depression study (www.star-d.org) has shown that approximately half of patients with nonpsychotic MDD respond to level 1 antidepressant treatment (citalopram), and only nearly 30% patients achieve remission. Theoretically, the cumulative remission rate of four sequential treatments is 67%, and the likelihood of achieving remission is high in the first two medication trials and then decreases. Thus, probing into the pathophysiology of MDD and customizing effective therapeutic strategies for every individual suffering from MDD are essential.

Endocrine system abnormalities, including abnormalities of the adrenal, gonadal, and thyroid axes, have been observed in depression for many decades, and the alterations in mood and cognition after treatment with endocrine function-targeting medications further suggest that hormones play an important role in the pathophysiology of MDD (Dwyer et al., 2020). The hypothalamic-pituitary-adrenal (HPA) axis is a crucial neuroendocrine system that controls stress reactions and orchestrates emotions and many other bodily processes (Dwyer et al., 2020). In particular, arginine-vasopressin (AVP), in addition to corticotropin-releasing factor (CRF), enhances the release of adrenocorticotropic hormones (ACTH) and participates in acute stress response (Rene et al., 2000; Lolait et al., 2007). The up-regulation of its receptor may contribute to maintaining corticotrophic responsiveness to chronic stress or depression (Rotondo et al., 2016). Early studies have found that cortisol concentrations in plasma and cerebrospinal fluid were elevated in MDD (Holsboer and Barden, 1996; Nemeroff, 1996) and that the frequency of the failure to respond to the dexamethasone suppression test was increased in patients with depression, despite the low sensitivity (about 44%) that limited its use as a diagnostic tool (Arana et al., 1985). Subsequent studies further observed blunted cortisol circadian rhythms (Stetler and Miller, 2011), excessive HPA axis activity (Amsterdam et al., 1987; Ising et al., 2007; Menke et al., 2016), and impaired negative feedback in MDD (Arborelius et al., 1999; Dwyer et al., 2020). Keller and colleagues found that high plasma cortisol was associated with worsened cognitive performance in patients with MDD and healthy controls and that patients with psychosis had higher cortisol level than healthy subjects and depressed patients without psychosis (Keller et al., 2017). Interestingly, patients with bipolar disorder (some of whom were in depressive episodes) had increased cortisol response to the combined dexamethasone/corticotrophin-releasing hormone test (Watson et al., 2004). Notably, HPA axis dysregulation and normalization failure after treatment are associated with poor clinical prognosis, including low response rates to antidepressants and high relapse and chronicity (Nelson and Davis, 1997; Young et al., 2004; Vreeburg et al., 2013).

On the basis of the abovementioned insights, some researchers have attempted to explore medications that modify HPA axis function for depression treatment. These medications include glucocorticoid (GR)/mineralocorticoid receptor (MR) antagonists, vasopressin receptor antagonists, and steroidogenesis inhibitors. However, the results of these clinical trials were mixed. For example, Jahn and his team (Jahn et al., 2004) found that metyrapone (a cortisol synthesis inhibitor) was effective as an adjunctive treatment for MDD, whereas another study (McAllister-Williams et al., 2016) had negative results. Similarly, evidence for the glucocorticoid receptor antagonist mifepristone was ambiguous (Watson et al., 2012; Block et al., 2018). Some medications had initial promising results but were then discontinued (Serradeil-Le Gal et al., 2005; Sanofi-Aventis, 2008), and several compounds had been abandoned due to their severe side effects or lack of efficacy (Binneman et al., 2008; Zorrilla and Koob, 2010; Dwyer et al., 2020). A previous meta-analysis that explored antiglucocorticoid and related treatments for psychosis assessed depression symptoms as secondary outcomes and found limited evidence (Garner et al., 2016). None of these strategies have been successfully translated into clinical use, and the efficacy and side effects of these strategies for the treatment of MDD remain uncertain. We therefore systemically searched available studies and conducted a meta-analysis to determine the effects and safety of HPA axis-based medications for MDD.

## Materials and Methods

By following the guidance of Preferred Reporting Items for Systematic Reviews and Meta-analysis, we prepared a study protocol with objectives, search strategy, participants, study type, outcome measurements, and data synthesis strategy for study organization and reporting (PROSPERO registration number: CRD42021247279).

### Search Methods

The PubMed and Web of Science electronic database were searched for all studies without date, publication type, or language limitations. The following terms and synonyms were used in [Title/Abstract]: (“CRFR1 antagonists” OR “GR antagonists” OR “MR agonists” OR “glucocorticoids” OR “cortisol synthesis inhibitors” OR “vasopressin receptor antagonists” OR “ketoconazole” OR “mifepristone” OR “fludrocortisone” OR metyrapone) AND (“mood disorders” OR “depression” OR “major depressive disorder” OR “major depressive disorder with psychotic symptoms” OR “bipolar depression” OR “treatment-resistant depression”) AND (“cortisol” OR “hypothalamic-pituitary-adrenal axis” OR “HPA”). Reference lists were also searched as a supplement. This search was completed on January 16, 2021.

### Study Selection Criteria and Quality Assessment

All relevant randomized controlled trials (RCTs) that compared drugs targeting the HPA axis with a placebo or other active treatments were included. Crossover studies and open-label trials that reported depression severity before and after treatment were also included. Reviews, case reports, comments, and animal or cell experimental studies were excluded. Study participants were required to be with MDD with or without psychotic symptoms and bipolar depression as defined by any diagnostic system. We excluded patients with other psychiatric comorbidities. When several studies reported a possibly overlapping population, the most complete study was included.

All included studies were rated for the risk of bias in accordance with the Cochrane Handbook for Systemic Reviews of Interventions (Higgins et al., 2011). The criteria assessed the quality of the clinical trials from six dimensions (eight items), including selection bias (e.g., allocation concealment), performance bias (e.g., blinding strategy), detection bias (e.g., outcome assessment), attrition bias (e.g., incomplete outcome data), reporting bias (e.g. selective reporting), and other biases. Two authors (Y.D. Ding and Z.R. Wei) independently inspected all searched articles. Rating was completed with Review Manager version 5.3. If any disagreement occurred, we discussed with each other or turned to the senior author (WB. Guo).

### Data Extraction and Outcome Measures

For each included study, we collected the following information: study region, study type, number of subjects, demographic and clinical characteristics of subjects, treatment strategy and conflict of interests (commercial sponsorship). In particular, for the continuous outcomes of RCTs, we extracted the mean and standard deviation (SD) of change data and depression scale scores endpoints. For the binary outcomes of RCTs, we extracted the number of responders and non-responders or the number of adverse events. We considered patients with a 50% reduction in depression scales as responders. For open-label trials, we extracted the mean and SD of depression scale scores at the baseline and after treatment. For crossover studies, we only extracted first-phase data given the potential bias of the carry-over effect. For studies with several treatment groups (such as different doses) or more than two relevant treatment arms, we presented treatment groups in additional comparisons. For studies without SDs reported, we first attempted to contact the authors; if the needed information was still unavailable, we calculated SDs from *p* values, confidence intervals (CIs), or other statistics in accordance with the methods provided by the Cochrane Handbook for Systemic Reviews of Interventions (Higgins et al., 2011).

The primary outcomes of this study were the average change and endpoint in depression severity scale scores. The secondary outcomes were response rate, the relative risk (RR) of side effects, and the average change in cognitive functioning scores.

### Statistical Analysis

The weighted mean differences or standardized mean differences (SMDs) and 95% CIs were used to compare continues outcomes. If the same measurement tool (e.g., the same depression symptom rating scale) was used to measure the same outcome in these included studies, the former was used. If not, then the latter one was used (Faraone, 2008). The RR and its 95% CIs were calculated to compare binary outcomes.

Statistical heterogeneity was inspected by using the *I* ^*2*^ method along with the *p* value from the chi-square test. Substantial heterogeneity was defined as *I* ^*2*^ ≥ 50% or *p* < 0.05. Given the potential inflation or deflation of the effect size caused by the random-effects model (Garner et al., 2016), the fixed-effects model was used for all analyses provided that substantial heterogeneity was absent between studies; otherwise, the random-effects model was applied. All the meta-analyses were done by using STATA SE version 12.0.

The publication biases of analyses including more than 10 studies were determined by using funnel plots or Egger’s test (Egger et al., 1997) with significance set at *p* < 0.05.

### Subgroup Analysis, Sensitivity Analysis, and Meta-regression

Subgroup analyses were conducted to examine the effect of different medications and the immediate, short-term and long-term effects of these medications.

If heterogeneity was high, we first performed influence analysis to investigate which study/studies had excessive influences on the result and excluded it/them and then performed re-analysis. We also conducted sensitivity analyses to investigate the possible variables contributing to the inconsistency of results. In other words, we assessed whether these variables changed the final conclusions (Thabane et al., 2013). We performed sensitivity analyses from the following aspects: 1) Study quality. We excluded studies with “high risk” and more than three “unclear risk” items. 2) Depression rating scale. We analyzed studies that used Hamilton Rating Scale for Depression (HAMD) and Montgomery-Asberg Depression Rating Scale (MADRS). 3) Treatment strategy. We examined the effects of HPA-axis-targeting medications used alone and as an add-on treatment. 4) Diagnosis. We analyzed the effect of these medications only for MDD (excluded studies that recruited patients with bipolar depression), treatment-resistant MDD and psychotic depression.

We explored the effect of age, the difference in the percentage of females between the intervention and control groups, and commercial sponsorship on study effect size by using meta-regression method (Huizenga et al., 2011). The random effects model that allowed for within- and between-study variations was chosen.

## Results

As shown in Supplementary Figure S1 (see Appendices), 1907 studies were included for title and abstract screening after 551 duplications were excluded. Subsequently, 38 studies were fully reviewed. A total of 23 publications that included 2,972 subjects were finally included in our analysis.

### Characteristics of the Included Studies

Table 1 and Supplementary Table S1 (Appendices) show the characteristics of the included studies and their treatment strategies. A total of 14 double-blind RCTs (Malison et al., 1999; Wolkowitz et al., 1999; Jahn et al., 2004; DeBattista et al., 2006; Flores et al., 2006; Binneman et al., 2008; Otte et al., 2010; Griebel et al., 2012; Watson et al., 2012; McAllister-Williams et al., 2016; GlaxoSmithKline, 2017; Katz et al., 2017; Block et al., 2018; Kamiya et al., 2020), one single-blind crossover study (O'Dwyer et al., 1995), one double-blind crossover study (Belanoff et al., 2001), and seven open-label studies were included (Thakore and Dinan, 1995; Dinan et al., 1997; Zobel et al., 2000; Belanoff et al., 2002; Rogoz et al., 2004; Simpson et al., 2005; Paslakis et al., 2011). Supplementary Figure S2 (Appendices) shows the risk of bias of the included studies. Seven open-label studies were rated “high risk” for their lack of randomization and blinding. For other biases, crossover design and small sample size were regarded as “high risk”. Some studies that lacked detailed information on randomization generation or allocation concealment or outcome assessor blinding were rated as “unclear risk”. Given that the studies by Block and colleagues (Block et al., 2018), Binneman and colleagues (Binneman et al., 2008) and NCT00733980 (GlaxoSmithKline, 2017) did not provide the scores of depression severity after interventions, they were only included in the meta-analysis for change data. One study (DeBattista et al., 2006) only reported outcomes as the number of responders and non-responders. Three studies used metyrapone alone or as an adjunctive treatment in patients with MDD with or without a treatment-resistant feature. Seven out of eight studies used mifepristone as a monotherapy or as an adjunctive treatment in patients with psychotic depression. Only Watson and colleagues recruited patients with bipolar depression. Four studies used ketoconazole alone for patients with MDD with or without a treatment-resistant feature. Studies investigating Vasopressin 1 B (V_1B_) receptor antagonists and corticotropin-releasing hormone (CRH) receptor antagonists all focused on MDD. The remaining two studies explored dexamethasone and fludrocortisone/spironolactone, respectively. HAMD was used by the majority of studies to gauge depressive severity. Almost half of these studies had commercial sponsorship.

**TABLE 1:** Characteristics of eligible studies.

Study	Region	Study design	Diagnosis	Subjects (total)	Administration	Scale/Endpoint	Direct conflict of interests (commercial sponsorship)
Metyrapone							
Jahn et al. (2004)	Germany	Double-blind, placebo-controlled	MDD (DSM-IV)	63 (ITT)	3 weeks, add-on treatment, metyrapone (1.0 g/d), placebo	HAMD-21 Day 35	No
McAllister-Williams et al. (2016)	United Kingdom	Double-blind, placebo-controlled	Treatment-resistant MDD (DSM-IV)	165 (ITT)	3 weeks, add-on treatment, metyrapone (1.0 g/d), placebo	MADRS Day 35	No
O’Dwyer et al. (1995)	United Kingdom	Single-blind placebo-controlled, crossover	MDD (DSM-III)	8 (ITT)	2 weeks, mixed therapy, metyrapone (1.5–3 g/d), placebo	HAMD-17 Day 14	No
Rogoz et al. (2004)	Poland	Open-label, no placebo	Treatment-resistant MDD (DSM-IV)	9 (completers)	6 weeks, add-on treatment, metyrapone (0.5 g/d), placebo	HAMD Day 42	No
Mifepristone							
Block et al. (2018)	United States	Double-blind, placebo-controlled	Psychotic depression (DSM-IV)	1,460 (ITT)	7 days, monotherapy, mifepristone (300, 600, 1200 mg/d), placebo	HAMD-24 Day 56	Yes
Watson et al. (2012)	New Zealand	Double-blind, placebo-controlled	Bipolar depression (DSM-IV)	60 (ITT)	7 days, add-on treatment, mifepristone (600 mg/d), placebo	MADRS Day 49	Yes
Belanoff et al. (2001)	United States	Double-blind, placebo-controlled, crossover	Psychotic depression (DSM-IV)	5 (ITT)	4 days, monotherapy, mifepristone (600 mg/d), placebo	HAMD Day 5	No
Flores et al. (2006)	United States	Double-blind, placebo-controlled	Psychotic depression (DSM-IV)	30 (ITT)	8 days, mixed therapy, mifepristone (600 mg/d), placebo	HAMD-21 Day 8	Yes
DeBattista et al. (2006)	United States of America	Double-blind, placebo-controlled	Psychotic depression (DSM-IV)	221 (ITT)	7 days, add-on treatment, mifepristone (600 mg/d), placebo	HAMD-24 Day 28	Yes
Belanoff et al. (2002)	United States	Open-label, no placebo^a^	Psychotic depression (DSM-IV)	30 (completers)	7 days, mixed therapy, mifepristone (50 mg/d, 600 mg/d, 1200 mg/d)	HAMD-21 Day 7	Yes
Simpson et al. (2005)	Egypt	Open-label, no placebo	Psychotic depression (DSM-IV)	20 (LOCF)	6 days, monotherapy, mifepristone (600 mg/d)	HAMD-21 Day 28	Yes
Ketoconazole							
Wolkowitz et al. (1999)	United States	Double-blind, placebo-controlled	MDD (DSM-IV)	20 (ITT)	4 weeks, monotherapy, ketoconazole (400–800 mg/d), placebo	HAMD-21 Day 28	No
Malison et al. (1999)	United States of America	Double-blind, placebo-controlled	Treatment-refractory MDD (DSM-III)	16 (LOCF)	6 weeks, monotherapy, ketoconazole (600–1200 mg/d), placebo	HAMD-19 Day 42	No
Thakore and Dinan. (1995)	United Kingdom	Open-label, no placebo	MDD (DSM-III)	8 (completers)	4 weeks, monotherapy, ketoconazole	HAMD-17 Day 28	No
Paslakis et al. (2011)	Germany	Open-label, no placebo	Treatment-resistant MDD, melancholic subtype (DSM-IV)	6 (completers)	3 weeks, monotherapy, ketoconazole (600 mg/d increased to 800 mg/d)	HAMD-21 Day 21	No
Vasopressin V_1B_ receptor antagonist							
Kamiya et al. (2020)	United States	Double-blind, placebo-controlled	MDD (DSM-V)	46 (ITT)	6 weeks, add-on treatment, TS-121 (10 mg/d and 50 mg/d), placebo	MADRS Day 56	Yes
Griebel et al. (2012)	Multinational	Double-blind, placebo-controlled	MDD (DSM-IV)	Study1: 218 (ITT) Study2: 233 (ITT)	Study1 and Study2: 8 weeks, monotherapy, SSR149415 (200 mg/d and 500 mg/d), placebo	HAMD-17 Day 56	Yes
Katz et al. (2017)	United States	Double-blind, placebo-controlled	MDD (DSM-IV)	51 (ITT)	7 days, monotherapy, ABT-436 (800 mg/d), placebo	HAMD-17 Day 8	Yes
CRH antagonist							
Binneman et al. (2008)	Multinational	Double-blind, placebo-controlled	Recurrent MDD (DSM-IV)	59 (interim analysis)	6 weeks, monotherapy, CP-316,311 (800 mg/d), placebo	HAMD-17 Day 42	Yes
NCT00733980	United States	Double-blind, placebo-controlled	MDD (DSM-IV)	150 (ITT)	6 weeks, monotherapy, GSK561679 (350 mg/d), placebo	HAMD-17 Day 42	Yes
Zobel et al. (2000)	Germany	Open-label, no placebo	MDD (DSM-IV)	20 (completers)	3 weeks, monotherapy, R121919 (5–40 mg/d, 40–80 mg/d)	HAMD-21 Day 30	Yes
Others							
Dinan et al. (1997)	United Kingdom	Open-label, no placebo	Treatment-resistant depression (DSM-III)	10 (ITT)	4 days, add-on treatment, dexamethasone (3 mg/d)	HAMD Day 21	No
Otte et al. (2010)	Germany	Double-blind, placebo-controlled	MDD (DSM-IV))	64 (ITT)	3 weeks, add-on treatment, fludrocortisone (0.2 mg/d), spironolactone (100 mg/d), placebo	HAMD-17 Day 21	No

a : Authors chose to use a 50-mg dose because the placebo response rate in psychotic major depression is very low. Although the dose of 50-mg/day dose does not appear to have significant antiglucocorticoid effects in humans, it still has antiprogesterone properties.

Abbreviation: CRH, corticotropin-releasing hormone; DSM, Diagnostic and Statistical Manual of Mental Disorders; HAMD, Hamilton Rating Scale for Depression; ITT, intention-to-treat; LOCF, last observation carried forward; MADRS, Montgomery-Asberg Depression Rating Scale; MDD, major depressive disorder.

Table 2 summarizes the demographic and clinical features of the included subjects. Most studies recruited both sexes. Only study NCT00733980 recruited exclusively women. The average age range was between 30 and 55 years, except in one study that included predominantly elderly subjects. Less than half of the included studies provided episode information.

**TABLE 2:** Demographic and clinical characteristics of the included subjects.

Study	Subjects	Women	Age	Inpatients	Previous episodes	Duration of current episode (months)
Jahn et al. (2004)	Metyrapone (*n* = 33)	54%	45.2 ± 13.8	100%	2	3
	Placebo (*n* = 30)	53%	46.5 ± 13.0	100%	2	3.5
McAllister-Williams et al. (2016)	Metyrapone (*n* = 83)	57%	47.6 ± 9.9	100%	—	—
	Placebo (*n* = 82)	63%	45.2 ± 10.4	100%	—	—
O’Dwyer et al. (1995)	Metyrapone (*n* = 4)	75%	40.3 ± 8.4	100%	—	—
	Placebo (*n* = 4)	100%	39.5 ± 7.5	100%	—	—
Rogoz et al. (2004)	*N* = 9	67%	52.4 ± 2.3	100%	6.4 ± 0.7	—
Block et al. (2018)	Mifepristone (*n* = 833)	58%	44.7 ± 11.6	Most were outpatients	—	—
	(300 mg n = 110, 600 mg *n* = 471, 1200 mg *n* = 252)	60%	44.7 ± 11.2		—	—
Women, age, ect.	Placebo (*n* = 627)					
Watson et al. (2012)	Mifepristone (*n* = 30)	50%	48 ± 9.3	0	—	17 ± 20
	Placebo (*n* = 30)	43%	48 ± 9.5	0	—	12 ± 18.8
Belanoff et al. (2001)	Mifepristone (*n* = 2)	0%	47.5 ± 3.5	100%	0	5.5 ± 3.5
	Placebo (*n* = 3)	67%	56 ± 11.5	100%	1	7.0 ± 9.5
Flores et al. (2006)	Mifepristone (*n* = 15)	60%	36.4 ± 13.2	—	—	—
	Placebo (*n* = 15)	53%	38.8 ± 12.9	—	—	-
DeBattista et al. (2006)	Mifepristone (*n* = 105)	47%	40.9 ± 10.8	—	—	—
	Placebo (*n* = 116)	52%	41.6 ± 11.0	—	—	—
Belanoff et al. (2002)	50 mg mifepristone (*n* = 11)	55%	42.3 ± 11.6	100%	—	3.7 ± 4.2
	600 mg + 1200 mg mifepristone (*n* = 19)	68%	47.0 ± 14.8	100%	—	4.3 ± 6.5
Simpson et al. (2005)	Mifepristone (*n* = 20)	30%	46.0 ± 10.8^a^	100%	—	—
Wolkowitz et al. (1999)	Ketoconazole (*n* = 9)	60% (total)	46.9 ± 14.0 (total)	0%	—	—
	Placebo (*n* = 11)			0%	—	—
Malison et al. (1999)	Ketoconazole (*n* = 8)	50%	44 ± 8	75%	—	—
	Placebo (*n* = 8)	25%	45 ± 14	62.5%	—	—
Thakore et al. (1994)	Ketoconazole (*n* = 8)	50%	42.7 ± 2.3	100%	—	—
Paslakis et al. (2011)	Ketoconazole (*n* = 6)	33%	66.8 ± 11.1	100%	1.5 ± 1.5	31.2 ± 22.0
Kamiya et al. (2020)	TS-121 10 mg (*n* = 14)	62.5%	44.8 ± 12.6	—	—	10.9 ± 15.1
	TS-121 50 mg (*n* = 15)	68.8%	44.8 ± 12.9	—	—	17.9 ± 25.6
	Placebo (*n* = 17)	66.7%	45.8 ± 11.1	—	—	24.9 ± 54.3
Griebel et al. (2012)		67.5%	41.0 ± 10.7	0%	—	—
	SSR 200 mg (*n* = 77)	50.6%	42.0 ± 11.0	0%	—	—
	SSR 500 mg (*n* = 70)	63.2%	41.2 ± 12.4		—	—
	Placebo (*n* = 71)	74.7%	43.0 ± 12.4		—	—
		70.7%	41.6 ± 11.9		—	—
	SSR 200 mg (*n* = 79)	73.4%	40.1 ± 10.6		—	—
	SSR 500 mg (*n* = 78)					
Women, age, ect.	Placebo (*n* = 76)					
Katz et al. (2017)	ABT-436 (*n* = 31)	35% (total)	35.5 ± 9.95 (total)	—	—	27.6 ± 50
	Placebo (*n* = 20)					14.9 ± 25
Binneman et al. (2008)	CP-316,311 (*n* = 28)	39%	50 ± 13.5	0%	—	—
	Placebo (*n* = 31)	35%	49 ± 11	0%	—	—
NCT00733980	GSK561679 (*n* = 74)	100%	38.8 ± 11.3	—	—	—
	Placebo (*n* = 76)	100%	40.8 ± 12.2	—	—	—
Zobel et al. (2000)	R121919 (*n* = 20)	45%	47.2 ± 12.2	—	—	4.5 ± 2.7
Dinan et al. (1997)	Dexamethasone (*n* = 10)	60%	35.4 ± 7.9	—	1.1 ± 1.3	4.0 ± 1.5
Otte et al. (2010)	Fludrocortisone (*n* = 24)	63%	36.5 ± 12.7	67%	1.5 ± 1.4	7.4 ± 5.4
	Spironolactone (*n* = 27)	61%	36.7 ± 10.6	63%	0.6 ± 0.9	8.0 ± 9.9
	Placebo (*n* = 13)	64%	34.5 ± 12.7	75%	0.8 ± 1.2	5.2 ± 3.3

### Primary Outcomes

**FIGURE 1:**
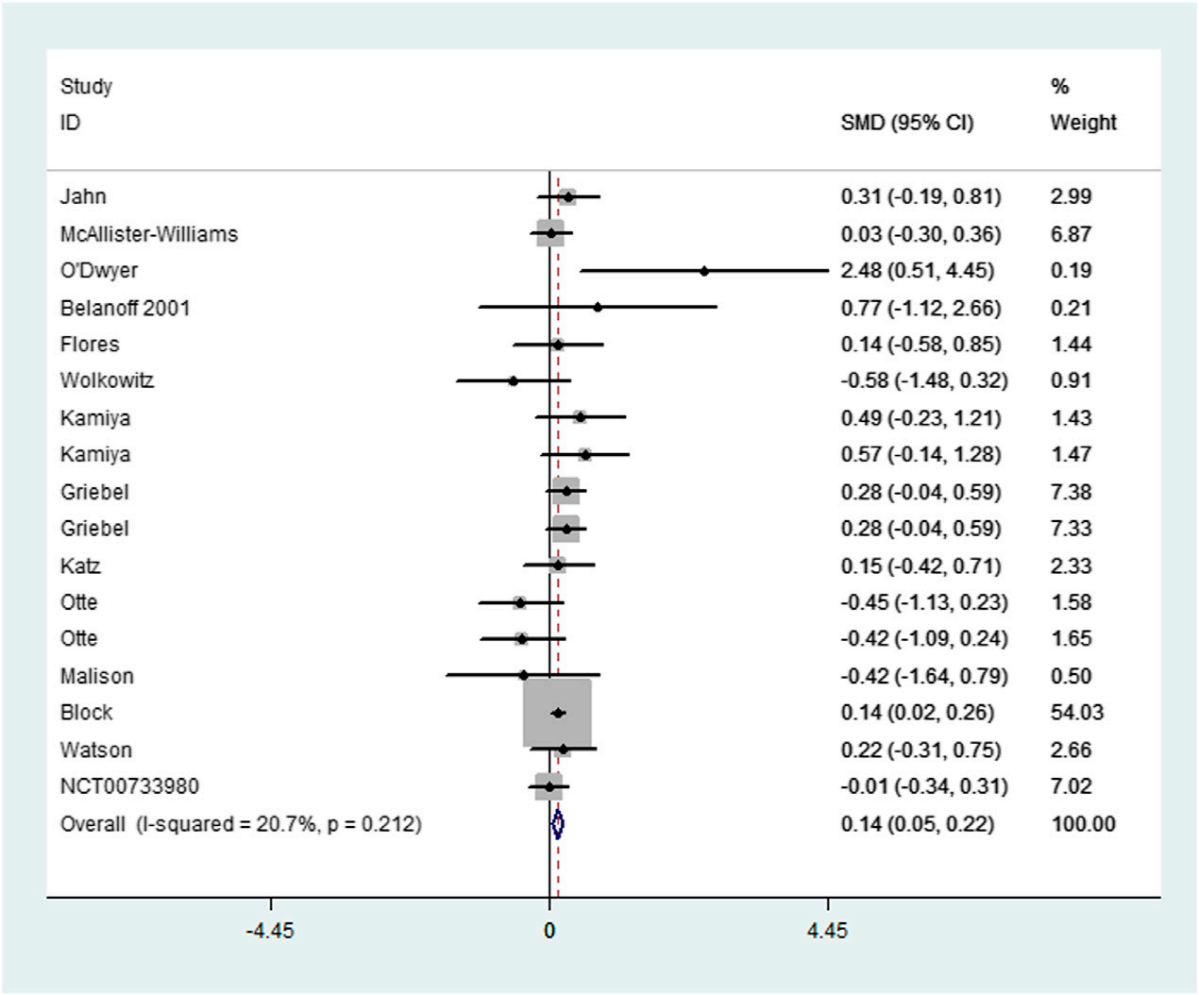
Forest plot and meta-analysis (change data) of the efficacy of HPA-targeting treatments for depression.

**FIGURE 2:**
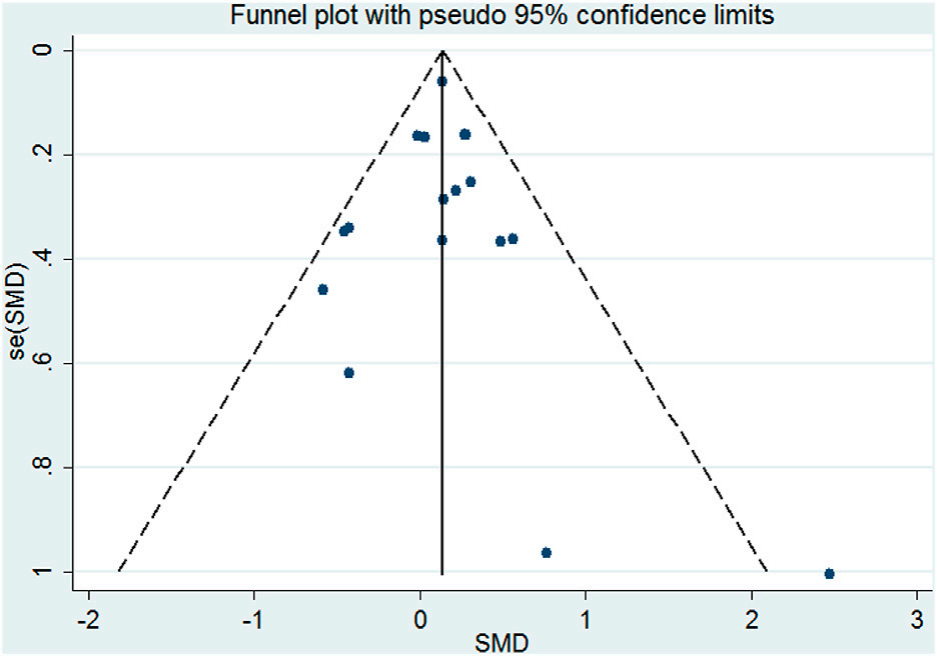
Funnel plots illustrating the meta-analysis (change data) of the efficacy of HPA-targeting treatments for depression.

As illustrated in Supplementary Table S2 (Appendices), pooling the change data of 20 comparisons that assessed the efficacy across all included HPA-targeting medications showed a significant difference between interventions and controls with very small heterogeneity after influence analysis (Figure 1, SMD = 0.138, 95%CI = 0.052, 0.224, *p* = 0.002; I^2^ = 20.7%, *p* = 0.212). No obvious publication bias was observed (Figure 2, *p* = 0.127). Subgroup analysis, which was performed to assess the efficacy of the different types of medications, revealed a significant difference favoring mifepristone and V_1B_ receptor antagonist treatment (SMD = 0.146, 95%CI = 0.033, 0.258, *p* = 0.011 and SMD = 0.404, 95%CI = 0.255, 0.533, *p* = 0). No heterogeneity was reported in both groups (*p* = 0.919 and *p* = 0.668). No significant difference was observed either in the metyrapone, ketoconazole, or CRH receptor antagonist group. The analysis of follow-up outcomes recorded by six studies 2 weeks after treatment discontinuation showed a difference in favor of interventions and no heterogeneity (SMD = 0.156, 95%CI = 0.053, 0.259, *p* = 0.003; I^2^ = 0%, *p* = 0.676). For the immediate effect of HPA-modulating treatments, a significant difference was observed but heterogeneity was high (I^2^ = 72.3%, *p* = 0), and the difference was not significant after influence analysis.

Sensitivity analysis for high-quality studies revealed that antiglucocorticoid treatment and related treatments had a significant effect for depression and low heterogeneity (SMD = 0.183, 95%CI = 0.102, 0.264, *p* = 0; I^2^ = 37.6%, *p* = 0.059). However, the publication bias was significant (*p* = 0.039). Sensitivity analysis for studies using HAMD, for patients with unipolar depression and for patients with psychotic depression also showed a significant difference favoring intervention (SMD = 0.131, 95%CI = 0.039, 0.223, *p* = 0.005; SMD = 0.136, 95%CI = 0.049, 0.223, *p* = 0.002; SMD = 0.141, 95%CI = 0.026, 0.257, *p* = 0.016) and moderate heterogeneity after influence analysis (I^2^ = 30.8%, *p* = 0.137; I^2^ = 25.4%, *p* = 0.168; I^2^ = 0%, *p* = 0.915). Sensitivity analysis for studies using HPA-modulating treatment alone showed a significant effect compared with controls with moderate heterogeneity after influence analysis (SMD = 0.288, 95%CI = 0.151, 0.426, *p* = 0; I^2^ = 42.3%, *p* = 0.086). However, when used as an add-on treatment, these interventions had a similar effect as controls (*p* = 0.418).

No change was observed in the significance of any of the outcomes in the meta-analysis for endpoint data, except for mifepristone subgroup analysis and sensitivity analysis for psychotic depression, which showed no difference between interventions and controls (*p* = 0.133, *p* = 0.373), and immediate effect analysis, which showed that HPA-modulating treatment had a significant immediate effect for depression compared with controls (SMD = -0.234, 95%CI = -0.375, -0.093, *p* = 0.001). We conducted a meta-analysis for seven open-label trials, and the results showed a significant reduction in depression scale scores after intervention; however, the heterogeneity was high (I^2^ = 93.4%, *p* = 0).

### Secondary Outcome

Meta-analysis for the three studies that recorded outcomes as responders and non-responders revealed similar efficacy between the interventions and controls (RR = 1.073, 95%CI = 0.892, 1.290, *p* = 0.455).

**FIGURE 3:**
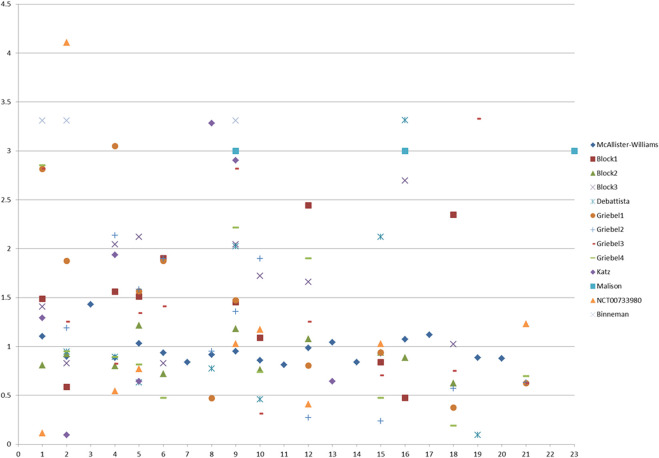
Scatter plot of the RRs of 23 side effects of HPA-targeting medications reported by eight included RCTs. RR, risk ratio.

Adverse events were reported by 14 studies. Our analysis (see Supplementary Table S3, Appendices) for all the studies and high-quality studies showed a significant difference in favor of controls (RR = 1.319, 95%CI = 1.165, 1.493, *p* = 0 and RR = 1.283, 95%CI = 1.134, 1.452, *p* = 0). Heterogeneity was moderate after influence analysis (I^2^ = 43.4%, *p* = 0.061 and I^2^ = 39.3%, *p* = 0.106). No publication bias was found (*p* = 0.832 and *p* = 826). Mifepristone had more adverse events than the controls. However, heterogeneity was high, and the difference was not significant after influence analysis (RR = 1.052, 95%CI = 0.981, 1.128, *p* = 0.157; I^2^ = 11.0%, *p* = 0.338). Analysis for V_1B_ receptor antagonists showed a significant difference in favor of controls, and no heterogeneity was observed (RR = 2.018, 95%CI = 1.414, 2.879, *p* = 0; I^2^ = 0, *p* = 0.726). In particular, patients under HPA-modulating treatment experienced more dizziness (RR = 1.289, 95%CI = 1.035, 1.604, *p* = 0.023) and dyspepsia/nausea/vomiting (after influence analysis, RR = 1.637, 95%CI = 1.296, 2.066, *p* = 0) than the controls. No heterogeneity was reported (*p* = 0.545 and *p* = 0.690). Figure 3 shows the RRs of the 23 side effects of the examined medications.

Only one trial (Watson et al., 2012) reported cognition function and no significant difference was reported (see Supplementary Table S4, Appendices).

### Meta-Regression

Our meta-regression analysis for all included studies and high-quality studies revealed that age and the difference in the percentage of females between the intervention and control groups had no significant effect on outcomes (*p* = 0.391 and *p* = 0.520, respectively). Similar results were reported for high-quality studies. However, in the analysis for high-quality studies, commercial sponsorship had a significant effect on outcomes and could explain 12.83% of between-study variance (*p* = 0.026).

## Discussion

This meta-analysis of 16 RCTs and seven open-label studies that included 2,972 subjects examined the efficacy and safety of HPA-targeting treatments for depression as a monotherapy or as an add-on treatment. The following medications were explored: metyrapone, mifepristone, ketoconazole, V_1B_ receptor antagonists (TS-121, SSR149415, and ABT-436), CFH receptor antagonists (CP-316,311, GSK561679, and R121919), dexamethasone, spironolactone, and fludrocortisone. Our results indicated the favorable but small effect of these medications as a whole in treating individuals with depression or MDD. Effectiveness was remained significant during the 2 weeks follow-up period. Sponsorship might affect the results to a small extent. Evidence for ketoconazole, dexamethasone and fludrocortisone was scant, whereas the efficacy of mifepristone and V_1B_ receptor antagonists was more convincing than that of other medications. In particular, the V_1B_ receptor antagonist revealed a medium effect size. Our findings yielded insufficient evidence to support the efficacy of HPA-modulating medications for cognitive impairment in patients with depression. Although the action of these medications as the sole treatment for patients with depression was promising, additional studies were needed to verify their efficacy as augmenting agents. Safety analysis favored placebos. The rate of gastrointestinal side effects and dizziness were higher in subjects using antiglucocorticoids and related medications than in subjects using placebos.

Mifepristone, a GR antagonist, has been one of the most tested HPA-targeting medications for depression or psychosis. A meta-analysis reported by Garner and her team concluded the possible efficacy of mifepristone for psychosis (Garner et al., 2016). In our analysis, almost all of the included mifepristone studies were performed with patients with psychotic depression. Previous studies suggested that hyperactivity of the HPA axis may lead to dopaminergic and noradrenergic dysregulation and dysfunction. For example, researchers have found that dexamethasone, adrenocorticotropin, and CRH boost dopamine metabolites (Rothschild et al., 1984; Posener et al., 1994; Posener et al., 1999). Duval and colleagues (Duval et al., 2006) found lower cortisol and growth hormone response to dopamine receptor agonists and alpha 2-adrenoreceptor agonists, respectively, in patients with psychotic major depression than in healthy controls and nonpsychotic patients. The function of somatodendritic and postsynaptic 5-hydroxytryptamine 1 A (5-HT_1A_) receptors is also regulated by corticosteroids and decreases in response to chronic stress (Grino et al., 1987; Guillaume et al., 1987; Laaris et al., 1997; Fairchild et al., 2003; McAllister-Williams et al., 2007). This situation may be the underlying mechanism of the efficacy of mifepristone used alone or with selective serotonin reuptake inhibitors for psychotic depression. However, the whole picture of this mechanism is complex and remains unclear. Interestingly, some researchers have hypothesized that the effectiveness of mifepristone may be associated with mifepristone plasma levels. One of the included studies (Block et al., 2018) examined this hypothesis and found a greater response rate in patients with high mifepristone plasma levels than in those without. Thus, the lack of significant difference for depression between mifepristone and placebos reported by some studies may be attributed to subtherapeutic mifepristone plasma levels. However, predicting plasma levels in accordance with dosage is difficult due to the nonlinear kinetics of mifepristone when administered at doses exceeding 50 mg (Sitruk-Ware and Spitz, 2003). Further studies may benefit from examining not only the level of mifepristone but also the levels of its active metabolites which increase reliably with dosage (Heikinheimo et al., 1987; Sitruk-Ware and Spitz, 2003). Some researchers have suggested that the efficacy of antiglucocorticoid treatment may be associated with baseline cortisol levels. Previous studies indicated that HPA disturbances, including abnormal basal and post-dexamethasone cortisol levels, are more likely to manifest in patients with psychotic depression than in non-psychotic depressed subjects (Contreras et al., 2007). Lombardo and colleagues conducted a meta-analysis (Lombardo et al., 2019) to investigate this hypothesis and found that patients who responded to cortisol synthesis inhibitors rather than GR antagonists had higher baseline cortisol levels than non-responders. The different pharmacological actions of these medications may be a confounding factor. The efficacy of mifepristone may also be associated with inflammation levels instead of with baseline cortisol levels given the evidence provided by a previous animal study by Zhang and colleagues (Zhang et al., 2018). Other researchers focused on GR antagonism and hypothesized that the cerebrospinal fluid levels of cortisol and mifepristone may affect the therapeutic effects of these drugs (Golier and Yehuda, 2018). Studies have suggested that mifepristone has a potent p-glycoprotein pump antagonist role that can control the transport of cortisol across the blood-brain barrier (Schatzberg and Lindley, 2008). These hypotheses await testing in the future.

Cortisol synthesis inhibitors, including metyrapone and ketoconazole, were also broadly examined in patients with MDD. In general, the quality of most studies examining these two medications was relatively low, and our results showed no benefit from treatment. Similarly, our results did not reveal the beneficial effect of either CRH receptor antagonists or MR-modulating treatments due to scarce evidence. CRH system hyperactivation had been reported in patients with MDD, and the abnormality was restored after antidepressant treatment (Nemeroff et al., 1991). Some clinical trials had explored related compounds for depression treatment but few are ongoing due to side effects or lack of efficacy (Dwyer et al., 2020). New compounds with different targets of the CRH system and different pharmacokinetic profiles may be useful (Dwyer et al., 2020). MR is another essential nuclear receptor that binds to cortisol with high affinity. It is mainly distributed in the prefrontal-limbic circuit (Dwyer et al., 2020). MR dysfunction was also found in depressed patients with equivocal results. Increases and decreases in MR function have been reported (Lopez et al., 1998; Lopez et al., 2003; Young et al., 2003; Wang et al., 2008). Thus, Otte and colleagues (Otte et al., 2010) examined the efficacy of the use of a MR antagonist (spironolactone) and a MR agonist (fludrocortisone) as an adjunct to escitalopram in patients with MDD. Although they found no significant difference in altered HAMD scores between interventions and controls, they observed that responders in the fludrocortisone group responded earlier than the controls and patients in the spironolactone group. Response time is an important index and should be assessed in future studies. Early response is linked to reduced mental health care costs and personal suffering. The fludrocortisone, according to (Otte et al., 2010), may play an accelerator role in depression, instead improving overall psychopathology. Existing evidence indicated the potential antidepressant effect of MR-modulation. For example, MR upregulation was one of the earliest response to antidepressants in several animal studies (Barden et al., 1995; Yau et al., 2002), which was also observed in studies on GR antagonists (Bachmann et al., 2003). The continued response (at least 2 weeks) to GR antagonists and cortisol synthesis inhibitors seen in our results may be partly mediated by MR up regulation and MR/GR balance resetting (Schatzberg and Lindley, 2008). In addition, MR stimulation led to increased 5-HT_1A_ receptor expression in mice (Rozeboom et al., 2007). While we cannot deny the possibility that some observed effects of fludrocortisone are exerted by GR stimulation, despite that its affinity for GR is much lower than for MR. On the contrary, evidence from MR antagonist spironolactone weas more controversial. Previously, (Holsboer, 1999), demonstrated adverse effects of spironolactone for depression treatment, and similarly, (Otte et al., 2010), found no improvement for depressed patients treated with spironolactone as an add-on strategy. However, other studies, for example studies focusing on patients with premenstrual syndrome (O'Brien et al., 1979; Wang et al., 1995) and euthymic patients with bipolar disorder (Juruena et al., 2009), have reported beneficial results of spironolactone on mood or residual symptoms improvement. Thus, MR-targeting medications are promising approaches in the future.

Three studies examining V_1B_ receptor antagonists were graded as high quality, and our results showed a medium effect size for significant improvement in depression symptoms. Early animal studies had found that vasopressin-containing neurons were distributed in limbic areas and that their messenger RNA expression was increased by chronic stress (Rabadan-Diehl et al., 1995; Hernando et al., 2001; Stemmelin et al., 2005). AVP was elevated in approximately 25% of patients with MDD (van Londen et al., 1997) and normalized after antidepressant treatment (De Bellis et al., 1993). Although the preliminary results were positive, the potential antidepressant efficacy of the V_1B_ receptor antagonist needs further repetition.

Overall, a higher rate of adverse events was observed in patients treated with HPA-targeting medications than in controls, and the quality of evidence was relatively high. No serious adverse events were reported, and few patients dropped out due to side effects. Thus, these medications were well-tolerated. Notably, given the homology between glucocorticoid receptors and progesterone receptors and the potential risk of inducing abortion and amenorrhea in females, current GR antagonists may benefit from the further development of receptor selection (Schatzberg and Lindley, 2008).

It is important to mention that glucocorticoid secretion is characterized by a complex, circadian and ultradian pattern which is under the influence of genes, age, gender and environments (Kalafatakis et al., 2016). This pulsatility has substantial, multi-level effects not only on the peripheral tissues, but also on the central nervous system. Recently, many researchers have found significant neurobiological effects of glucocorticoid ultradian rhythm on human, including cognitive, emotional and behavioral processes (Kalafatakis et al., 2018; Kalafatakis et al., 2019; Kalafatakis et al., 2021). Different cortisol replacement regimens resulted in varying outcomes, and subjects with regimen that failed to mimic the ultradian profile had lower health-related quality of life (Bleicken et al., 2010; Tiemensma et al., 2014; De Bucy et al., 2017). It seems like few studies in this meta-analysis have considered this issue, and most of these studies had simplified drug administration strategies. No study to date has elucidated the effects of different treatment regimens on depression, and it remains unclear whether treatment outcomes improve if HPA-modulating drugs are administered according to cortisol ultradian rhythmicity. Given the complexity and variability of glucocorticoid pulsatility and its neurobiological significance, it is a great challenge for future studies to optimize the application and therapeutic evaluation of HPA axis-modulating therapies for MDD. In addition, it is also noteworthy that even though many preclinical and clinical studies suggest a strong association of HPA-axis in stress induced mental disorders like depression and posttraumatic stress disorder. There is also evidence that not all depressed patients do display alterations of the HPA axis. A HPA-axis dysregulation biomarker is not employed clinically in routine settings. Therefore, it is unclear whether such treatments can benefit all patients or not. This study suggests that patients with psychotic depression and treatment-resistant MDD may be benefit from HPA axis examination and HPA axis-modulating therapy. However, more clinical trials are warranted to this issue.

One main limitation of this research is the variable quality of the included studies, which may bias our results. Many of them have “unclear risk of bias” items due to the lack of necessary information, such as randomization, allocation, or blinding procedures. Additional well-designed RCTs with prolonged follow-up periods investigating HPA-modulating treatments in different depression subtypes are needed. In addition to the change in depression rating scores, other outcome measures, such as response rate, remission rate, time to respond, and functional improvement along with cognitive function must be investigated. These measures are more understandable and feasible for clinical use than other measures. Simultaneously, the measurement of the plasma level of medications, peripheral and central neuroendocrine indexes, and inflammation factors may be useful for establishing optimal doses and understanding underlying mechanisms. Given the remarkable sex difference in the HPA system (Kokras et al., 2019), patient stratification may be useful for diminishing confounding factors. In addition, head-to-head trials are also important to determine whether these treatments are a better choice for patients with MDD than standard treatments.

## Conclusion

In conclusion, this is the first meta-analysis examining the efficacy and safety of HPA-targeting medications in patients with MDD. Our results suggest the favorable but small effect of these medications as a whole for MDD. Specifically, evidence for mifepristone and V_1B_ receptor antagonists are more convincing than that for other medications. Although adverse effect analysis favors placebos, these drugs are generally well-tolerated. HPA-based medications are a promising field for depression treatment, but additional high-quality RCTs, including head-to-head trials, are needed to verify findings.

## Data Availability

The original contributions presented in the study are included in the article/Supplementary Material, further inquiries can be directed to the corresponding author.
