# Supplementary material for: Efficacy of Treatments Targeting Hypothalamic-Pituitary-Adrenal Systems for Major Depressive Disorder: A Meta-Analysis
Source: Front Pharmacol. 2021 Sep 10;12:732157. doi: 10.3389/fphar.2021.732157 (PMC8461240; doi:10.3389/fphar.2021.732157)

Table S1 Treatment strategy of the included studies.

| Study | Standard treatment |
| --- | --- |
| Jahn et al. 2004 | Fluvoxamine (150-200 mg/d) or nefazodone (300-400 mg/d). |
| McAllister-Williams et al. 2015 | Participants continued their existing antidepressant regimen. |
| O’Dwyer et al. 1995 | Six patients were drug-free, the others were maintained on their constant treatment regimen. Metyrapone dosage was titrated according to plasma cortisol, the initial dosage was 500mg qid and the maximal dosage was 1g qid. |
| Rogoz et al. 2004 | Imipramine 0.1g/d for 6 weeks and then metyrapone was added and administered jointly with imipramine for further 6 weeks. |
| Block et al. 2018 | Patients had not been taking antidepressants or antipsychotics for 7days (30 days for fluoxetine) prior to enrollment. |
| Watson et al. 2012 | Several antidepressants, mood stabilizers and antipsychotics were used. |
| Belanoff et al. 2001 | Patients were required not to take antipsychotic medication for 3 days before the trial. No patients were taking antidepressants when entering the study. |
| Flores et al. 2006 | Antidepressants, antipsychotics and mood stabilizers were allowed to use, 10% were drug-free or used anxiolytics alone. |
| Debattista et al. 2006 | Antipsychotics and antidepressants were not allowed for at least 7 days prior to and during the study. These drugs and electroconvulsive therapy were allowed to use after day 7. |
| Belanoff et al. 2002 | Patients were allowed to remain on their stable antidepressants or/and antipsychotic medications. Drug-free patients were also included. |
| Simpson et al. 2005 | Except lorazepam, no other psychotropic medications were used for at least 1 week prior to the trial. |
| Wolkowitz et al. 1998 | Patients had drug-free for at least 6 weeks. Seven of them had never been treated with antidepressants. |
| Malison et al. 1999 | Patients had a minimum 2-week washout period from all psychotropic medications and terfenadine. |
| Thakore et al. 1994 | Six of patients were drug-free and two had been drug-free for at least 6 weeks. |
| Paslakis et al. 2011 | All included patients were used at least two antidepressants before entered the study. After admission, patients were kept off these drugs for at least 6 days prior to the study period. |
| Kamiya et al. 2020 | Patients were required to have remained on the single antidepressant for at least 6 weeks, with a fixed dose for at least 4 weeks prior screening, and to have had an inadequate response to the current therapy. |
| Griebel et al. 2012 | Patients who have used the following prior to entry into Acute Phase were exclued: antipsychotics within 3 months, fluoxetine within 1 month, MAOIs within 2 weeks, other antidepressants, anxiolytics, or mood-stabilizer (lithium, anticonvulsants) within 1 week. |
| Katz et al. 2016 | Subjects had not used any other medication within 4 weeks prior to the trial. |
| Binneman et al. 2008 | The study included an initial 1-week single-blind placebo treatment phase and a 6-week fixed-dose, double-blind, double-dummy, parallel-group treatment phase. |
| NCT00733980 | Patients who had taken other psychoactive drugs within 1 week prior to the trial were excluded. |
| Zobel et al. 2000 | During the screening period all psychoactive drugs were stopped for a minimum of 5 days. Patients using hepatic enzyme inducers were not included. Pretreatment with monoamine oxidase inhibitors, fluoxetine or slow-release neuroleptics had to be discontinued for at least 1 month and treatment with corticosteroids or electroconvulsion within 3 months were not allowed. |
| Dinan et al. 1997 | Four patients treated with fluoxetine (40-60mg/d) and six with sertraline (100-200mg/d). |
| Otte et al. 2010 | Escitalopram (10mg/d started, could be increased during the following weeks)  The concomitant use of lorazepam and/or zolpidem/zopiclon was allowed, whereas any use of antipsychotics or mood stabilizers was not permitted. |

Table S2 Results of the meta-analysis of RCTs and open-label trials.

| Group | comparison (s) | Subjects | ES (*p*) | 95%CI | *I^2^* (*p*) | P value of Egger’s test |
| --- | --- | --- | --- | --- | --- | --- |
| **Change data** | | | | | | |
| Total | 20 | 2835 | 0.162 (0) | 0.082, 0.242 | 62.1% (0) | 0.132 |
| Influence analysis | 17 | 2558 | 0.138 (0.002) | 0.052, 0.224 | 20.7% (0.212) | 0.127 |
| Subgroup1: | | | | | |  |
| Metyrapone | 3 | 214 | 0.361 (0.263) | -0.271, 0.993 | 67.9% (0.044) | - |
| Influence analysis | 2 | 206 | 0.116 (0.407) | -0.158, 0.390 | 0% (0.363) | - |
| Mifepristone | 4 | 1547 | 0.146 (0.011) | 0.033, 0.258 | 0% (0.919) | - |
| Ketoconazole | 2 | 36 | -0.523 (0.156) | -1.247, 0.200 | 0% (0.838) | - |
| Vasopressin V1B receptor antagonist | 7 | 548 | 0.404 (0) | 0.255, 0.553 | 0% (0.668) | - |
| CRH receptor antagonist | 2 | 205 | -0.278 (0.051) | -0.557, 0.001 | 90% (0.002) | - |
| Others | 2 | 64 | -0.437 (0.073) | -0.915, 0.041 | 0% (0.955) | - |
| Subgroup2: | | | | | | |
| Immediate effect | 14 | 850 | 0.171 (0.008) | 0.045, 0.297 | 72.3% (0) | 0.180 |
| Influence analysis | 11 | 573 | 0.098 (0.216) | -0.057, 0.254 | 40.0% (0.082) | 0.522 |
| Short-term effect (2w) | 6 | 1764 | 0.156 (0.003) | 0.053, 0.259 | 0% (0.676) | - |
| Short-term effect (≥ 6w) | 2 | 1512 | -0.036 (0.599) | -0.171, 0.099 | 0% (0.916) | - |
| Sensitivity analysis1: | | | | | | |
| High quality studies | 17 | 2542 | 0.183 (0) | 0.102, 0.264 | 37.6% (0.059) | 0.039 |
| Sensitivity analysis2: | | | | | | |
| MADRS | 4 | 241 | 1.743 (WMD) (0.109) | -0.391, 3.877 | 0% (0.456) | - |
| HAMD | 16 | 2646 | 0.159 (0) | 0.074, 0.243 | 68.3% (0) | 0.131 |
| Influence analysis | 13 | 2369 | 0.131 (0.005) | 0.039, 0.223 | 30.8% (0.137) | 0.113 |
| Sensitivity analysis3: | | | | | | |
| Add-on treatment | 7 | 368 | 0.104 (0.418) | -0.148, 0.357 | 31% (0.192) | - |
| Monotherapy | 11 | 2208 | 0.170 (0) | 0.082, 0.258 | 72.0% (0) | 0.148 |
| Influence analysis | 9 | 689 | 0.288 (0) | 0.151, 0.426 | 42.3% (0.086) | - |
| Sensitivity analysis4: | | | | | | |
| Unipolar depression | 19 | 2783 | 0.161 (0) | 0.080, 0.241 | 64.0% (0) | 0.142 |
| Influence analysis | 16 | 2506 | 0.136 (0.002) | 0.049, 0.223 | 25.4% (0.168) | 0.138 |
| Treatment-resistant MDD | 2 | 181 | 0.003 (0.983) | -0.313, 0.320 | 0% (0.480) | - |
| Psychotic depression | 3 | 1495 | 0.141 (0.016) | 0.026, 0.257 | 0% (0.915) | - |
|  | | | | | | |
| **Endpoint** | | | | | | |
| Total | 17 | 949 | -0.250 (0) | -0.369, -0.131 | 30.5% (0.113) | 0.136 |
| Subgroup1: | | | | | | |
| Metyrapone | 3 | 214 | -0.441 (0.194) | -1.106, 0.224 | 70.5% (0.034) | - |
| Mifepristone | 3 | 87 | -0.325 (0.133) | -0.749, 0.099 | 0% (0.844) | - |
| Ketoconazole | 2 | 36 | 0.511 (0.174) | -0.226, 1.247 | 51.8% (0.150) | - |
| Vasopressin V1B receptor antagonist | 7 | 548 | -0.335 (0) | -0.484, -0.187 | 0% (0.783) | - |
| Others | 2 | 64 | 0.294 (0.225) | -0.181, 0.769 | 0% (0.817) | - |
| Subgroup2: | | | | | | |
| Immediate effect | 12 | 645 | -0.234 (0.001) | -0.375, -0.093 | 41.5% (0.065) | 0.097 |
| Short-term effect (2w) | 5 | 304 | -0.288 (0.011) | -0.509, -0.067 | 1.7% (0.397) | - |
| Short-term effect (≥ 6w) | 1 | 52 | -0.063 (0.822) | -0.606, 0.481 | - | - |
| Sensitivity analysis1: | | | | | | |
| High quality studies | 15 | 936 | -0.243 (0) | -0.363, -0.124 | 20.3% (0.227) | 0.037 |
| Sensitivity analysis2: | | | | | | |
| MADRS | 4 | 241 | -2.130 (WMD) (0.071) | -4.441, 0.181 | 27% (0.250) | - |
| HAMD | 13 | 708 | -0.247 (0) | -0.383, -0.112 | 37.7% (0.083) | 0.080 |
| Sensitivity analysis3: | | | | | | |
| Add-on treatment | 7 | 368 | -0.201 (0.125) | -0.459, 0.056 | 32.3% (0.181) | - |
| Monotherapy | 8 | 543 | -0.268 (0.001) | -0.419, -0.117 | 16.1% (0.303) | - |
| Sensitivity analysis4: | | | | | | |
| Unipolar depression | 16 | 897 | -0.245 (0) | -0.367, -0.124 | 34.6% (0.086) | 0.149 |
| Treatment-resistant MDD | 2 | 181 | -0.072 (0.654) | -0.389, 0.244 | 0% (0.864) | - |
| Psychotic depression | 2 | 35 | -0.305 (0.373) | -0.975, 0.366 | 0% (0.606) | - |
|  | | | | | | |
| **Open-label trial** | 7 | 103 | 13.807 (0) | 10.972, 16.642 | 93.4% (0) | - |
|  | | | | | | |
| **Response rate** | 3 | 380 | 1.073 (RR) (0.455) | 0.892, 1.290 | 0% (0.674) | - |

Abbrevations: CI, confidential interval; CRH, corticotropin-releasing hormone; ES, effect size; HAMD, Hamilton Rating Scale for Depression; MADRS, Montgomery-Asberg Depression Rating Scale; MDD, major depressive disorder; WMD, weighted mean difference; RR, risk ratio.

Table S3 Results of the meta-analysis for safety.

| Adverse effect (s) | Comparison (s) | Subjects | RR (*p*) | 95%CI | *I^2^* (*p*) | P value of Egger’s test |
| --- | --- | --- | --- | --- | --- | --- |
| Total | 15 | 2619 | 1.141 (0) | 1.081-1.205 | 55.0% (0.005) | 0.353 |
| Influence analysis | 11 | 1525 | 1.319 (0) | 1.165-1.493 | 43.4% (0.061) | 0.832 |
| Sensitivity analysis: | | | | | | |
| High quality | 13 | 2544 | 1.133 (0) | 1.073-1.196 | 55.7% (0.008) | 0.334 |
| Influence analysis | 9 | 1450 | 1.283 (0) | 1.134-1.452 | 39.3% (0.106) | 0.826 |
| Subgroup analysis: | | | | | | |
| Metyrapone | 1 | 143 | 1.273 (0.05) | 1.0-1.622 | - | - |
| Mifepristone | 5 | 1711 | 1.103 (0.001) | 1.042-1.166 | 60.1% (0.040) | - |
| Influence analysis | 4 | 1459 | 1.052 (0.157) | 0.981-1.128 | 11.0% (0.338) | - |
| Vasopressin V1B receptor antagonist | 7 | 548 | 2.018 (0) | 1.414-2.879 | 0% (0.726) | - |
| CRH antagonist | 2 | 209 | 0.869 (0.374) | 0.637, 1.185 | 59.5% (0.116) | - |
|  | | | | | | |
| Types of side effects | | | | | | |
| Nervousness/Anxiety/ Agitation | 10 | 2244 | 1.106 (0.194) | 0.950-1.287 | 0% (0.775) | 0.234 |
| Decreased/Increased sleep | 12 | 2535 | 0.919 (0.386) | 0.758-1.113 | 0% (0.754) | 0.241 |
| Tremor | 1 | 143 | 1.430 (0.128) | 0.902-2.267 | - | - |
| Headache | 11 | 2476 | 1.146 (0.056) | 0.997-1.317 | 72.5% (0) | 0.848 |
| Influence analysis | 8 | 1610 | 1.129 (0.323) | 0.888-1.435 | 45.2% (0.078) | - |
| Dizziness | 11 | 2476 | 1.289 (0.023) | 1.035-1.604 | 0% (0.545) | 0.078 |
| Drowsiness | 8 | 2054 | 0.966 (0.751) | 0.779-1.198 | 0% (0.655) | - |
| Myoclonus | 1 | 143 | 0.838 (0.580) | 0.557-1.259 | - | - |
| Abdominal pain/Abdominal discomfort | 5 | 633 | 0.895 (0.578) | 0.605-1.324 | 0% (0.891) | - |
| Dyspepsia/ Nausea/Vomiting | 13 | 2551 | 1.447 (0) | 1.277-1.639 | 53.3% (0.012) | 0.200 |
| Influence analysis | 10 | 1685 | 1.637 (0) | 1.296-2.066 | 0% (0.690) | 0.364 |
| Diarrhea/ Constipation | 11 | 2476 | 1.083 (0.358) | 0.914-1.284 | 66.1% (0.001) | 0.821 |
| Influence analysis | 7 | 1559 | 0.911 (0.604) | 0.642-1.294 | 34.7% (0.163) | - |
| Decreased/increased appetite | 1 | 143 | 0.812 (0.153) | 0.611-1.080 | - | - |
| Weakness/Fatigue | 9 | 2204 | 1.095 (0.335) | 0.911-1.317 | 39.7% (0.103) | - |
| Sweating/Flushing | 2 | 194 | 1.035 (0.770) | 0.821-1.305 | 0% (0.617) | - |
| Postural hypotension | 1 | 143 | 0.838 (0.395) | 0.557-1.259 | - | - |
| Dry mouth | 9 | 2204 | 1.063 (0.537) | 0.875-1.292 | 39.3% (0.106) | - |
| Oedema/Rash | 6 | 1840 | 1.385 (0.092) | 0.948-2.022 | 29.1% (0.217) | - |
| Blurred vision | 1 | 143 | 1.121 (0.643) | 0.691-1.819 | - | - |
| Back pain/Myalgia | 7 | 1911 | 0.815 (0.312) | 0.548-1.212 | 36.7% (0.149) | - |
| Reproductive system and breast disorders | 3 | 582 | 0.792 (0.706) | 0.236-2.661 | 30.3% (0.238) | - |
| Weight gain/loss | 1 | 143 | 0.877 (0.571) | 0.558-1.380 | - | - |
| Infections and infestation | 5 | 601 | 0.702 (0.073) | 0.477-1.033 | 0% (0.891) | - |
| Toothache | 1 | 221 | 6.629 (0.078) | 0.811-54.156 | - | - |
| Metallic taste | 1 | 16 | 3.000 (0.482) | 0.140-64.262 | - | - |

Abbreviations: CI, confidence interval; CRH, corticotropin-releasing hormone; RR, risk ratio.

Table S4 Results of the meta-analysis for cognitive function.

| Study | Cognition | ES (p) | 95%CI |
| --- | --- | --- | --- |
| Watson et al. 2012 | SWM between search errors | -0.471 (0.094) | -1.022-0.081 |
|  | Rey 1 | 0 (1.0) | -0.544-0.544 |
|  | Rey 2 | -0.029 (0.917) | -0.572-0.515 |
|  | Visuo-spatial learning and memory (pattern) | -0.296 (0.288) | -0.843-0.250 |
|  | Visuo-spatial learning and memory (spatial) | 0.034 (0.901) | -0.509-0.578 |
|  | Short-term memory span | -0.157 (0.572) | -0.701-0.388 |
|  | Executive function (verbal fluency test) | 0.189 (0.497) | -0.356-0.734 |
|  | Executive function (backward digit span) | -0.073 (0.794) | -0.616-0.471 |
|  | Attention | -0.229 (0.410) | -0.775-0.316 |

Abbreviations: CI, confidence interval; ES, effect size; SWM, spatial working memory; Rey, the Rey-Auditory Verbal Learning Test; Rey 1, recall of list A after five repetitions, distraction and a delay; Rey 2, the total number of words recalled after five repetitions of list A.

Figure S1 PRISMA flow diagram of study selection


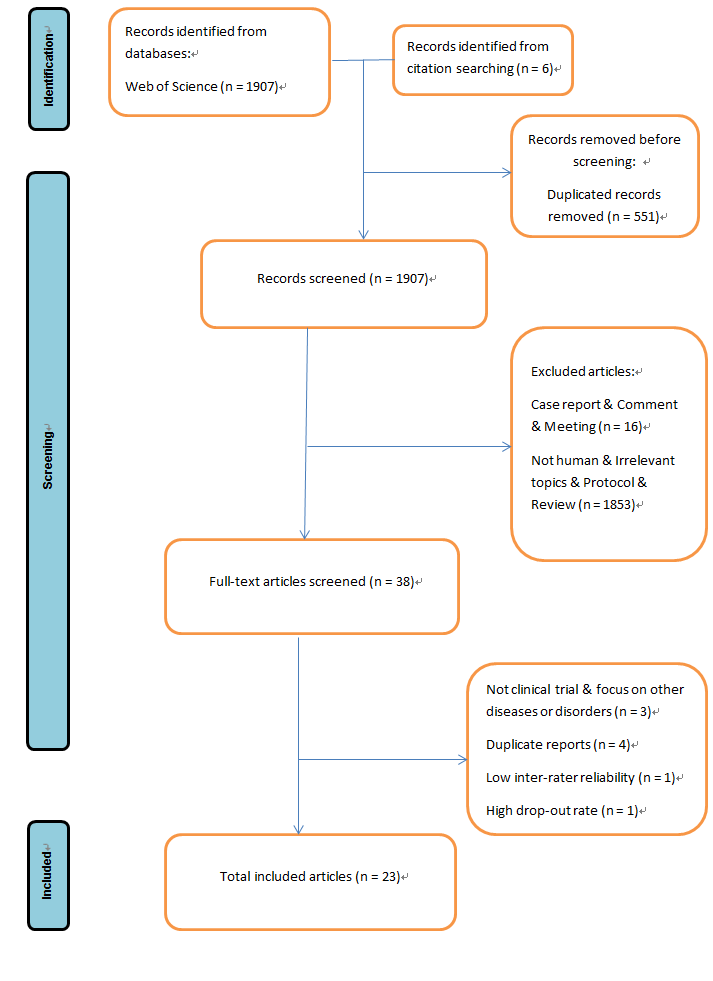


Figure S2 Risk of bias summary: review authors’ judgements about each risk of bias item for each included study.


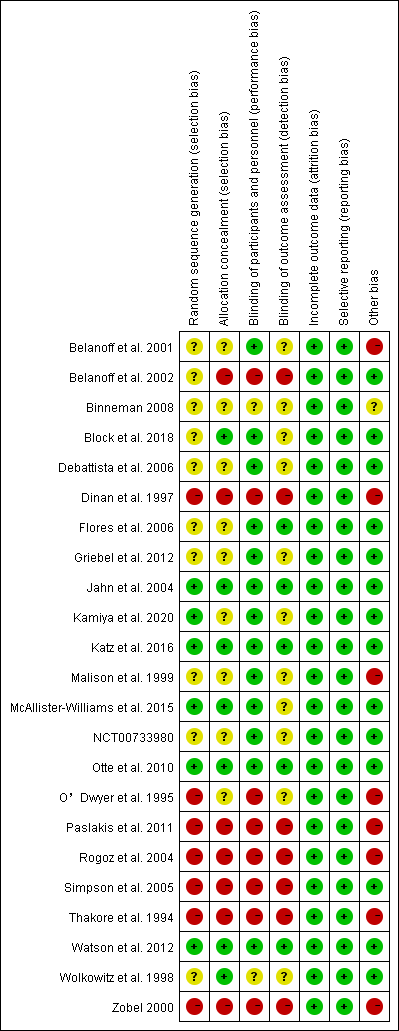

Supplement: Supplementary file 1 [file DataSheet1.DOCX]
